# Free-living marine nematodes community structure in the conservation area (Chaojing Park) and its adjacent area of Keelung, Taiwan

**DOI:** 10.1371/journal.pone.0268691

**Published:** 2022-05-27

**Authors:** Wei-Ling Ng, Cheng-Ann Chen, Saleem Mustafa, Chen-Lin Soo, Yun-Chih Liao, Tung-Wei Shih

**Affiliations:** 1 Borneo Marine Research Institute, Universiti Malaysia Sabah, Kota Kinabalu, Sabah, Malaysia; 2 Institute for Tropical Biology and Conservation, Universiti Malaysia Sabah, Kota Kinabalu, Sabah, Malaysia; 3 Department of Earth and Life Science, University of Taipei, Taipei, Taiwan (R.O.C.); 4 National Museum of Marine Science & Technology, Keelung, Taiwan (R.O.C.); Kaohsiung Medical University, TAIWAN

## Abstract

Studies conducted in the same seas or even study sites nearby each other, showed very different community structure, implying the patchiness of free-living marine nematodes which may be related to the sedimentary environment such as sediment type and food availability of the study area. This study was motivated by the concerns about the impacts of high level of anthropogenic activities on Chaojing Park (gazetted as Wanghaixiang’s Chao-Jing Bay Resource Conservation Area (WCJBRA) in 2016). The present study provides baseline knowledge of free-living marine nematode community structure in WCJBRA and identify potential marine nematodes as bioindicators to indicate possible impacts of the anthropogenic activities to the Chaojing Park. A total of 15 stations were selected in the subtidal zones of WCJBRA and its adjacent area. Marine nematode sample collection was carried out on the 13th and 14th of September 2019 using SCUBA diving technique. Results showed positive correlation between nematode density and medium sand (500μm-1.0mm). Presence of certain species such as *Daptonema* sp., *Pomponema* sp. and *Innocuonema* sp. indicates presence of disturbances in S12 and S13. Several species also showed potential to be introduced as indicator for healthy environment subjected to further studies on nematode-pollutants relationship, particularly on autecology as per se. Higher species diversity, H’ index of S1-S8 and S11 was categorised as Good Condition; followed by station with moderate species diversity index (S9, S10, S14 –Moderate Condition) zone; and lastly S12, S13 and S15 (Poor Condition).

## Introduction

Free-living marine nematodes exist as epifauna (on the sediment) or infauna (in the sediment) [1]. They comprise a sizable percentage of marine benthic populations, accounting for four out of every five bottom-dwelling metazoans on the world [2]. One of its ecological functions is to regulate nitrogen remineralization [3] by grazing on decomposers and generating ammonium for use by plants and bacteria [1,4]. Additionally, they are capable of immobilising carbon inside their body structure and re-mineralizing it during energy production via respiration or decomposition [1]. Additionally, marine nematodes contribute to the marine food cycle by acting as prey for predators [1,3,4].

The majority of studies on free-living marine nematodes were ecological investigations [5–7] or environmental pollution impact studies [7,8]. These are due to their close association with sediment, short life cycle, high reproduction rate, abundance, and small size [9–12], allowing them to respond quickly and early in the history of the contamination site [7,13–15]. As a result, investigations conducted in the same oceans or even close study sites revealed highly diverse community structures, emphasising the patchiness of free-living marine nematodes that may be related to the sedimentary environment, such as sediment type and food availability of the study area. For example, Liu [16] found 232 species and 149 genera in the community structure of nematode in the southern Yellow Sea. In the southern Taiwan Straits, there are 75 genera of free-living marine nematodes [17] and 101 species (58 genera) in Hong Kong waters [18].

Keelung City Government designated Chaojing as Wanghaixiang’s Chao-Jing Bay Resource Conservation Area (WCJBRA) in 2016. The conserved area was to ensure the survival of coral reefs as well as to address overfishing issues [19]. Although the bottom substrate was mainly comprise of rock, siltation was reported in Chaojing area, suffocating the coral reefs [20]. Moreover, Lin [20] also stated that sea urchin and lobsters which are indicator organisms for overfishing in the Reef Check Survey were rarely spotted in the area. As a result, significant concerns had been raised about the anthropogenic activity in the bay, as well as the presence of a commercial fishing harbour right close to the conservation area. The discharge of domestic garbage from surrounding restaurants, houses, boats, and shipping activities at the commercial fishing port, as well as antifouling paint activities in the harbour, may have an impact on the marine protected area’s environmental health. Few studies on free-living marine nematodes in relation to the impact of anthropogenic disturbance from fishing harbours have been conducted [21–23], and they have revealed that the meiofauna community responds well to anthropogenic disturbances.

Although free-living marine nematodes have shown promise in quantifying environmental status [7,8], it is unfortunate that little research has been conducted on the community structure of free-living marine nematodes in Taiwan, especially in marine protected area. The most recent study conducted was in northwest Taiwan on the intertidal zone of Danshuei River estuary [24]. Thus, this is the first study of its kind undertaken in Taiwan on the community structure of free-living marine nematodes at WCJBRA and its adjacent area, providing a solid foundation for future research. The aims of this study are to (1) characterise the community structure of free-living marine nematodes in the WCJBRA and nearby area, and (2) determine the physico-chemical environmental variables that influence the community structure of marine nematodes in this area.

## Methodology

**Fig 1:**
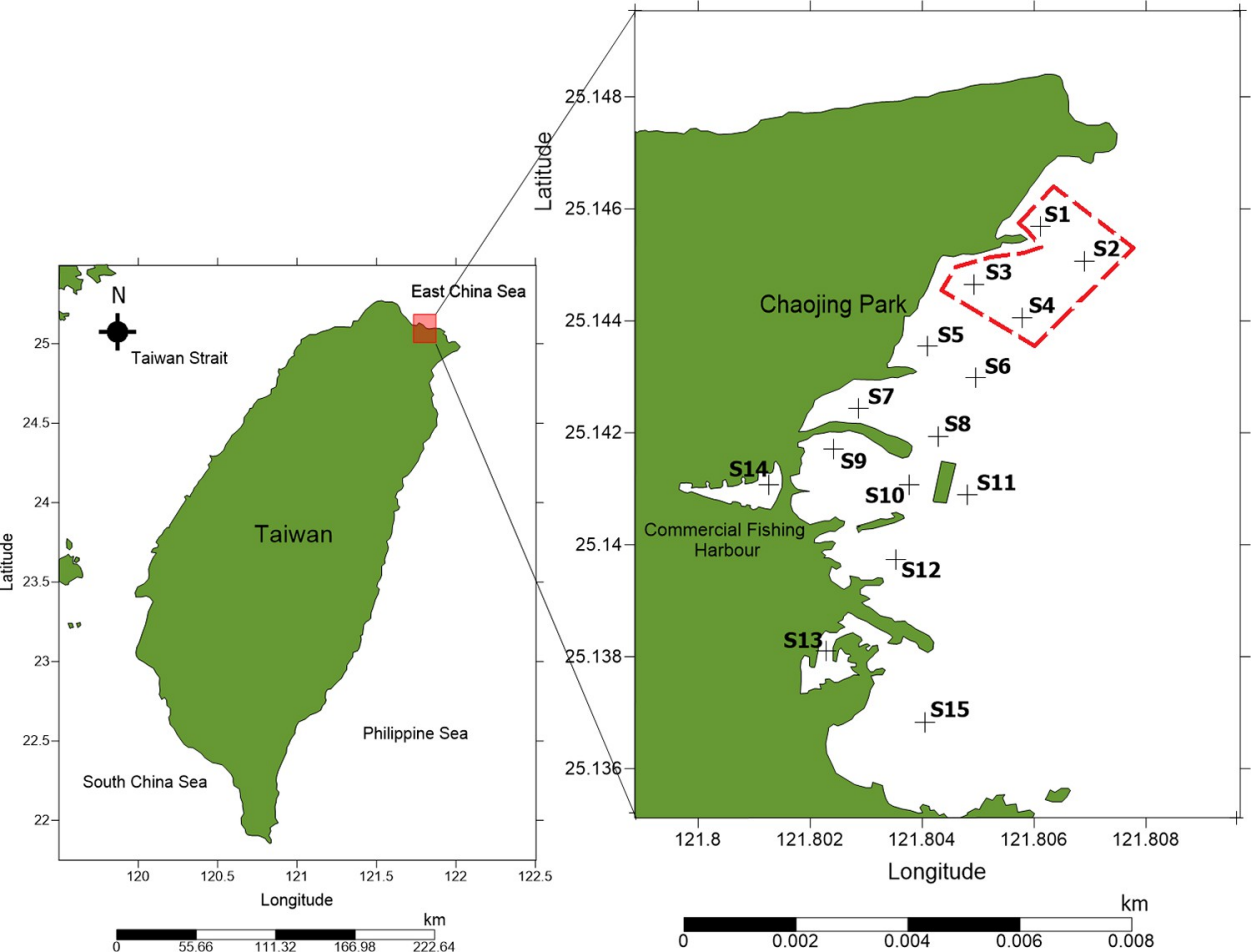
A total of 15 sampling sites with WCJBRA highlighted in red dashed line. Map illustrated by digitizing the base map of OpenStreetMap (under Open Database Lisence). Details refer to www.opendatacommons.org/licenses/odbl).

On the 13th and 14th of September 2019, this project was carried out in the subtidal zones of the marine conservation area and its surrounding region in Chaojing Park, Keelung, Taiwan. There was no historical environmental data in this location, but anthropogenic activity such as a fishing harbour, restaurants, antifouling coating, and a housing community were seen. A total of 15 sampling stations were chosen, and the geographical coordinates for each are shown below in Table 1. In Fig 1, the WCJBRA was denoted by a red dashed line, whereas the fishing harbour was at S14.

**Table 1:** Sampling regime and GPS coordination in Chaojing Park, Keelung, Taiwan.

Stations	GPS Coordinates		Sampling Date	Temperature (°C)	Salinity (ppt)	pH	DO (mg/L)	Remarks
1	N 25^o^ 8’ 43.4076"	E 121^o^ 48’ 21.6468"	13 Sept 2019	24.17±0.26	33.49±0.38	7.33±0.27	3.80±0.09	WCJBRA; sunny weather
2	N 25^o^ 8’ 42.4464"	E 121^o^ 48’ 23.382"	13 Sept 2019	23.33±0.52	34.86±0.11	7.26±0.05	5.50±0.09	WCJBRA; sunny weather
3	N 25^o^ 8’ 39.8616"	E 121^o^ 48’ 17.172"	13 Sept 2019	24.17±0.26	34.30±0.23	7.59±0.09	3.80±0.09	WCJBRA; sunny weather
4	N 25^o^ 8’ 38.7996"	E 121^o^ 48’ 19.9296"	13 Sept 2019	23.33±0.52	34.41±0.32	7.43±0.10	5.37±0.05	WCJBRA; sunny weather
5	N 25^o^ 8’ 34.494"	E 121^o^ 48’ 14.6376"	13 Sept 2019	24.33±0.52	32.34±0.51	7.05±0.12	5.43±0.14	Adjacent to WCJBRA; sunny weather
6	N 25^o^ 8’ 33.2844"	E 121^o^ 48’ 17.0496"	13 Sept 2019	24.17±0.26	33.52±0.45	7.10±0.09	4.10±0.27	Adjacent to WCJBRA; sunny weather
7	N 25^o^ 8’ 30.5484"	E 121^o^ 48’ 12.6432"	13 Sept 2019	24.67±0.52	34.34±0.26	7.39±0.09	4.54±0.13	Nearby to restaurants; sunny weather
8	N 25^o^ 8’ 28.9716"	E 121^o^ 48’ 15.678"	13 Sept 2019	24.83±0.26	34.69±0.24	7.29±0.23	3.70±0.09	Near to the harbour; sunny weather
9	N 25^o^ 8’ 28.5756"	E 121^o^ 48’ 10.2384"	13 Sept 2019	25.17±0.26	34.14±0.11	7.45±0.05	4.30±0.09	Near to the harbour; sunny weather
10	N 25^o^ 8’ 26.196"	E 121^o^ 48’ 13.8132"	13 Sept 2019	25.33±0.52	34.25±0.19	7.19±0.15	4.20±0.09	Near to the harbour; sunny weather
11	N 25^o^ 8’ 25.71"	E 121^o^ 48’ 17.6832"	14 Sept 2019	24.33±0.52	16.55±0.45	7.06±0.11	4.10±0.09	Near to the harbour; cloudy weather
12	N 25^o^ 8’ 20.7672"	E 121^o^ 48’ 13.8204"	14 Sept 2019	24.83±0.26	16.89±0.08	7.67±0.03	4.10±0.09	Near to the harbour; cloudy weather
13	N 25^o^ 8’ 15.2592"	E 121^o^ 48’ 8.8776"	14 Sept 2019	24.67±0.52	33.76±0.22	7.49±0.07	4.70±0.09	Small-scale harbour with few residentials; cloudy weather
14	N 25^o^ 8’ 26.5596"	E 121^o^ 48’ 5.3784"	13 Sept 2019	25.33±0.31	33.77±0.18	6.37±0.10	5.93±0.05	Commercial fishing harbour; sunny weather
15	N 25^o^ 8’ 10.8852"	E 121^o^ 48’ 15.354"	14 Sept 2019	27.80±0.24	33.97±0.23	7.04±0.05	4.65±0.04	Near to parking lot; rainy weather

The collection of marine nematode samples was done using the subtidal zone quantitative approach provided by Somerfield and Warwick [25]. Subtidal sediments have been gathered via SCUBA diving directly. A modified corer (11.95cm^2^) was used to gather triplicate samples. It was inserted vertically into sediments around 5cm deep. Additional sediment samples for particle size analysis have been obtained (PSA). Physico-chemical water quality parameters of bottom waters including salinity, temperature, dissolve oxygen (DO) and pH were measured *in situ* using a YSI multiparameter probe (ProPlus).

### Sample processing

Particle size samples were dried in an oven at 80°C for 24 hours to remove moist. Each sample weighing 100g was transferred to a beaker. Hydrogen peroxide were added to remove organic matter and left to stand overnight with sodium hexametaphosphate prior to dry sieving [26] to determine the particle size of the sediment from each station. Sediment retained on each sieve was weighed. Size of the sediments was graded according to the size scale suggested by [27].

Sediments collected for nematode analysis were sieved through a 500 μm mesh and decanted over a 25 μm mesh (Retch, ASTM E11) with tap water to remove macrofauna and microfauna prior to preservation for future extraction [23,28]. Sediments were washed into sample bottles and then preserved with 5% formalin [11]. Rose Bengal (0.5 g L^-1^) was applied and allowed to dye the nematode specimens thoroughly overnight. Sediments were rinsed and transferred to a centrifugation tube, which was filled to the 80% mark with Ludox and centrifuged at a rate of 3000 rpm for 5 minutes at 4°C. [29]. Supernatants were preserved in 5% formalin [23] for further sorting, counting, and identifying. The nematodes were isolated using a wire loop and dehydrated in ethanol-glycerol solution according to [30] to create permanent mounts for identification under a compound microscope using the nematode pictorial keys [31–36]. Additionally, marine nematodes have been divided into four trophic functional feeding groups (FFGs) based on their buccal cavity morphology: selective deposit feeders are designated as 1A; non-selective deposit feeders are designated as 1B; epigrowth feeders are designated as 2A; and predators/omnivores are designated as 2B [37–40].

### Data and statistical analyses

PRIMER v6 and SPSS (Statistical package for Social Science) v27 software were used for the statistical analyses in present study. DIVERSE analysis was used to determine the biological indices including average number of genera per sample (S), Total number of individuals (N), Shannon-Weiner Index (H’), Pielou’s Evenness Index (J’) and Margalef’s Index (d) from each station. The Maturity Index (MI) [41,42] was calculated as the weighted average of the individual colonizer-persistent (c-p) values to identify sites under stress. Threshold for nematode descriptors can be referred in Moreno’s study [13].Analysis of Similarity (ANOSIM) was used to determine the similarity of nematode communities between stations while Similarity Percentage (SIMPER) was used to identify the similarity percentage within and among each station. Multidimensional Scaling (MDS) with Bray-Curtis Similarity matric was used to illustrate the level of similarity of nematode species composition between stations. The data was square root transformed prior to the analysis. Nematode genera were classified according to Wieser [37] into four feeding groups: selective feeder (1A), nonselective feeder (1B), deposit feeders or epistrate feeders (2A), and predators or omnivores (2B) to investigate the trophic structure of the assemblages. CLUSTER analysis subjected to Euclidean distance was used to show the similarity of environmental parameters between stations. Pearson Correlation between environmental parameters and nematode density were conducted.

## Results

### Nematode composition and density

Across all sample sites in this research, a total of 111 nematode species from 25 families were identified and documented. DIVERSE analysis found that the northern study sites (S1–S10) had a higher biological indices value, while the southern research sites had a lower value (S11-S15). The maximum nematode density was found in S10 (N = 363 ± 91.65 ind./10cm^2^), while the lowest was found in S13 (N = 11 ± 8.89 ind./10cm^2^), followed by S12 (N = 15 ± 4.16 ind./10cm^2^). Stations situated inside WCJBRA, such as S2 (N = 115 ± 19.86 ind./10cm^2^) and S3 (N = 92 ± 17.95 ind./10cm^2^), had significantly higher nematode densities than other stations, but S1 and S4 had significantly lower densities (N = 50 ± 11.5 ind./10cm^2^ and N = 43 ± 4.36 ind./10cm^2^, respectively). On the other hand, S2 has the third largest total number of individuals (N), the most species (S = 31 ± 3.51), the most species richness (d = 6.19 ± 0.53), the highest species diversity (H’ = 3.11 ± 0.07%), and the second highest species evenness (J’ = 0.91 ± 0.01%). Additionally, S13 had the fewest species and individuals (S = 3 ± 2.08%; N = 11 ± 8.89 individuals/10cm^2^), the lowest species richness and diversity (d = 0.73 ± 0.25%; H’ = 0.79 ± 0.29%), but moderate high in species evenness (J’ = 0.87 ± 0.11%). In general, the nematode population in this research was dispersed uniformly (J’>0.7), except for S15 (J’ = 0.50 ± 0.33). Additionally, S15 has the second-lowest species richness and lowest diversity of species (d = 0.97 ± 0.30; H’ = 0.79 ±0.51%).

### Nematode assemblages

S4 had the lowest MI value (1.59 ± 0.05) and S13 had the highest (4.34 ± 0.20), followed by S15 (3.17 ± 0.22). Despite its location inside the WCJBRA, S4 was classified as having a bad environmental state using Moreno’s (13) standards. On the other hand, S14, the location of the commercial fishing port, had excellent environmental conditions. C-p 5 (extreme persisters) was not detected in all stations, while c-p 1 (extreme colonisers) was detected only in S12 (6.06%), which was dominated by c-p 2 (45.65%) and c-p 3. (37.10%). C-p 2 (general colonisers) and C-p 3 were typically dominant in all studied areas except for S13, S14 and S15 (Table 2). The lowest percentages of c-p 2 (11.85%) and c-p 3 (9.59%) were identified in S13 and S15 respectively. S14 were dominated by c-p 4 (53.55%) with lowest percentage of c-p 2 at 12.57%. The highest c-p 4 value were 58.55% in S15 (provided by *Eurystomina* sp.2). S4 had the greatest concentration of c-p 2 at 73.94%, which was mostly attributed to *Spilophorella* sp. (27.62%) and *Axonolaimus* sp. (21.23%). Whilst, the highest concentration of c-p 3 were identified in S8 at 59.98%, contributed by *Parapinnanema* sp. (13.32%) and *Innocuonema* sp. (10.81%).

**Table 2:** Maturity Index and c-p values (%) calculated for the assemblages of each study sites.

Faunal parameters	C-p 1	C-p 2	C-p 3	C-p 4	C-p 5	cp1 and cp2	cp3 to cp5	MI
S1	0.00	47.55	33.55	18.90	0.00	47.55	52.45	2.71 ± 0.05
S2	0.00	53.29	34.72	11.98	0.00	53.29	46.71	2.59 ± 0.04
S3	0.00	34.85	44.13	21.02	0.00	34.85	65.15	2.86 ± 0.09
S4	0.00	73.94	19.50	6.56	0.00	73.94	26.06	1.59 ± 0.05
S5	0.00	55.49	32.31	12.20	0.00	55.49	44.51	2.57 ± 0.06
S6	0.00	50.61	40.26	9.13	0.00	50.61	49.39	2.59 ± 0.05
S7	0.00	29.77	52.74	17.49	0.00	29.77	70.23	2.88 ± 0.06
S8	0.00	34.36	59.98	5.66	0.00	34.36	65.64	2.71 ± 0.08
S9	0.00	34.25	48.83	16.92	0.00	34.25	65.75	2.83 ± 0.08
S10	0.00	42.97	38.51	18.52	0.00	42.97	57.03	2.76 ± 0.12
S11	0.00	48.02	41.68	10.30	0.00	48.02	51.98	2.62 ± 0.06
S12	6.06	45.65	37.10	11.19	0.00	51.71	48.29	2.53 ± 0.09
S13	0.00	11.85	68.89	19.26	0.00	11.85	88.15	4.34 ± 0.20
S14	0.00	12.57	33.88	53.55	0.00	12.57	87.43	3.41 ± 0.16
S15	0.00	31.86	9.59	58.55	0.00	31.86	68.14	3.17 ± 0.22

The results of one-way ANOSIM revealed a 0.1% significant difference among groups with a R value of 0.748. SIMPER analysis showed that the marine nematode population in S10 had the highest percentage of similarity (58.47%), followed by S2 (55.44%) and then S14 (54.56%). Similar percentage in S10 was contributed by *Spirinia* sp. (26.6%), *Microlaimus* sp. (24.82%), and *Pomponema* sp. (10.24%) while S2 was contributed by *Axonolaimus* sp., *Metadesmodora* sp. and *Meyersia* sp. (9.95%, 6.91% and 6.63% respectively). On the other hand, the lowest similarity percentage within station was S11 for 20.45%. Most of the stations were highly dissimilar with S12, S13, and S15 in the community structure. In general, S12 has the highest dissimilarity percentage with S3 (96.82%), contributed by *Spilophorella* sp (12.23%)., *Draconema* sp. (11.23%), and *Acticonema* sp. (6.03%) whereas S13 has the highest dissimilarity with S1 (97.30%) on *Spillophorella* sp., *Viscosia* sp., and *Chromadorita* sp., (5.98%, 5.6% and 5.56% respectively). Besides that, *Eurystomina* sp.2 (16.06%), *Innocuonema* sp. (5.26%), and *Axonolaimus* sp. (5.18%) contributed the highest dissimilarity percentage between S7 and S15 with average dissimilarity of 97.95%. *Eurystomina* sp. 2 was recorded to be the dominant species in S15 was noted to have high dissimilarity percentage with other stations. Furthermore, S14 has the highest average dissimilarity with S15 (96.40%) due to the present of *Eurystomina* sp. (21.78%) and *Axonolaimus* sp. (7.8%) in S15, while *Bathyeurystomina* sp. with contribution of 12.14% in S14.

**Fig 2:**
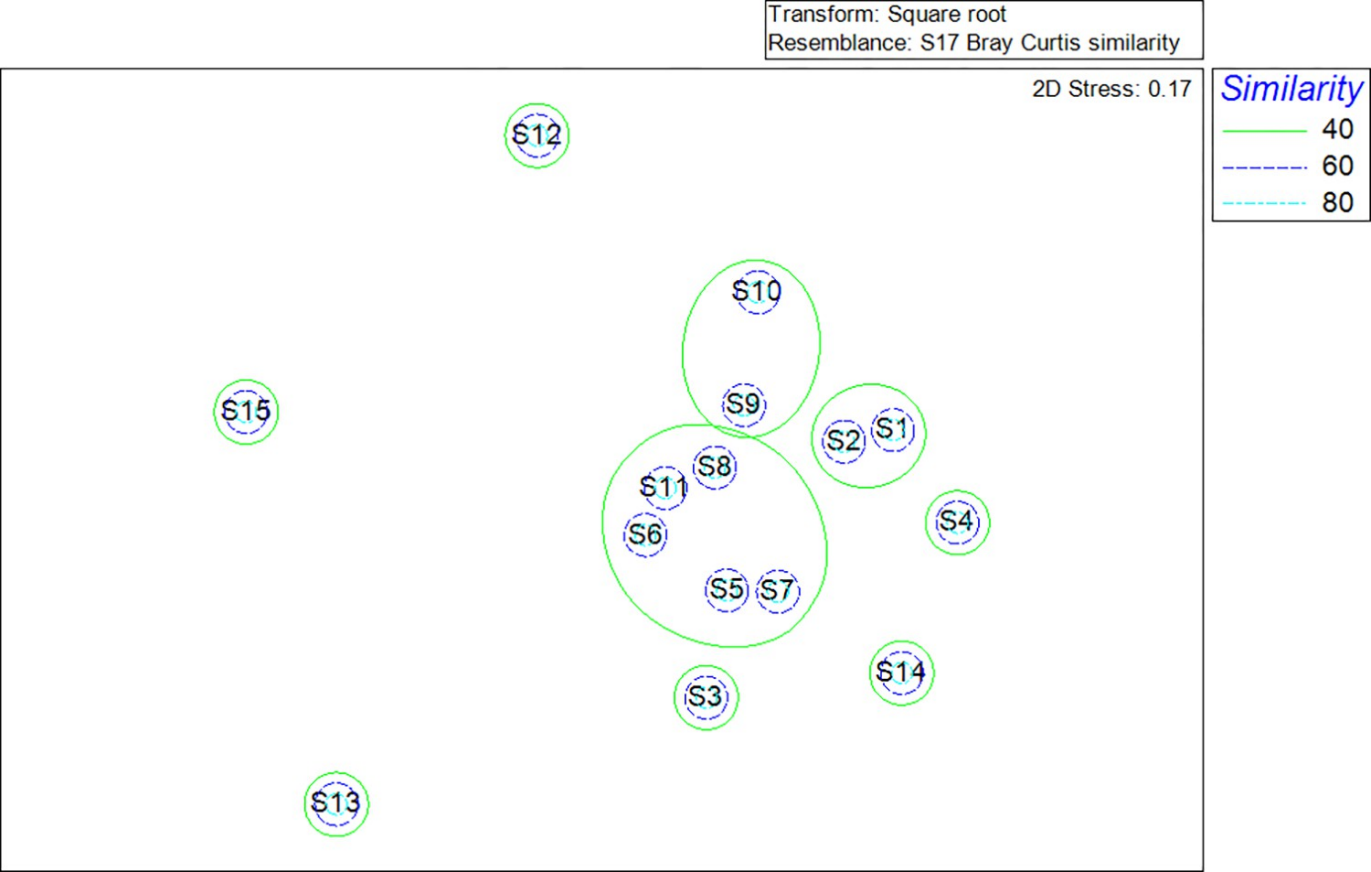
Multidimensional Scaling (MDS) illustrated the level of similarity of nematode species composition between stations.

The result of Multi-dimensional Scaling (MDS) on the species composition after square root transformation and Bray-Curtis similarity matrices revealed that S1 and S2 were grouped together at 40% similarity, followed by S5, S6, S7, S8, and S11, and S9 and S10. The remaining six stations were grouped independently as an independent group (Fig 2). Nonetheless, when the similarity threshold was raised to 60%, all stations formed autonomous groups. In general, the findings indicated a low degree of resemblance between the marine nematode communities at the research sites. Each cluster has its own distinct mix of nematode species.

**Fig 3:**
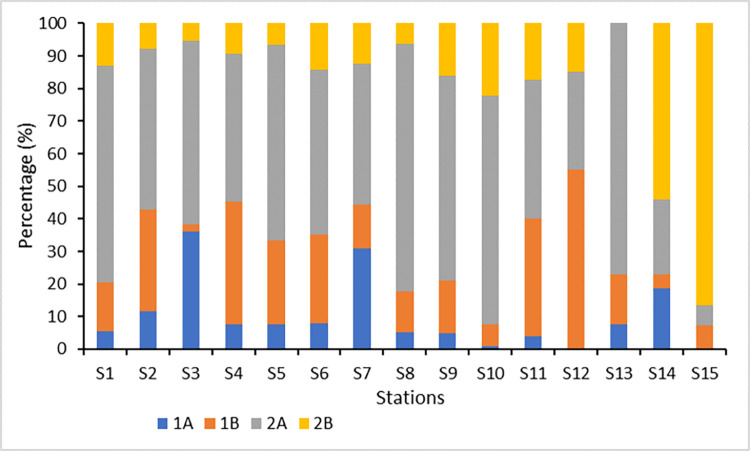
Percentage of Functional Feeding Group (FFG) of marine nematodes in the conservation area (Chaojing Park) and its adjacent area of Keelung, Taiwan.

Fig 3 shows the percentage of FFG in each station where most of the stations were dominated by 2A except S12, S14 and S15. In fact, S12 was dominated by 1B (55.00%) while both S14 and S15 were comprised mostly of 2B (S14: 54.17%; S15: 86.50%). On the other hand, the highest percentage of 2A was found in S13 at 76.92%.

### Sedimentary characteristics and Pearson correlation

**Fig 4:**
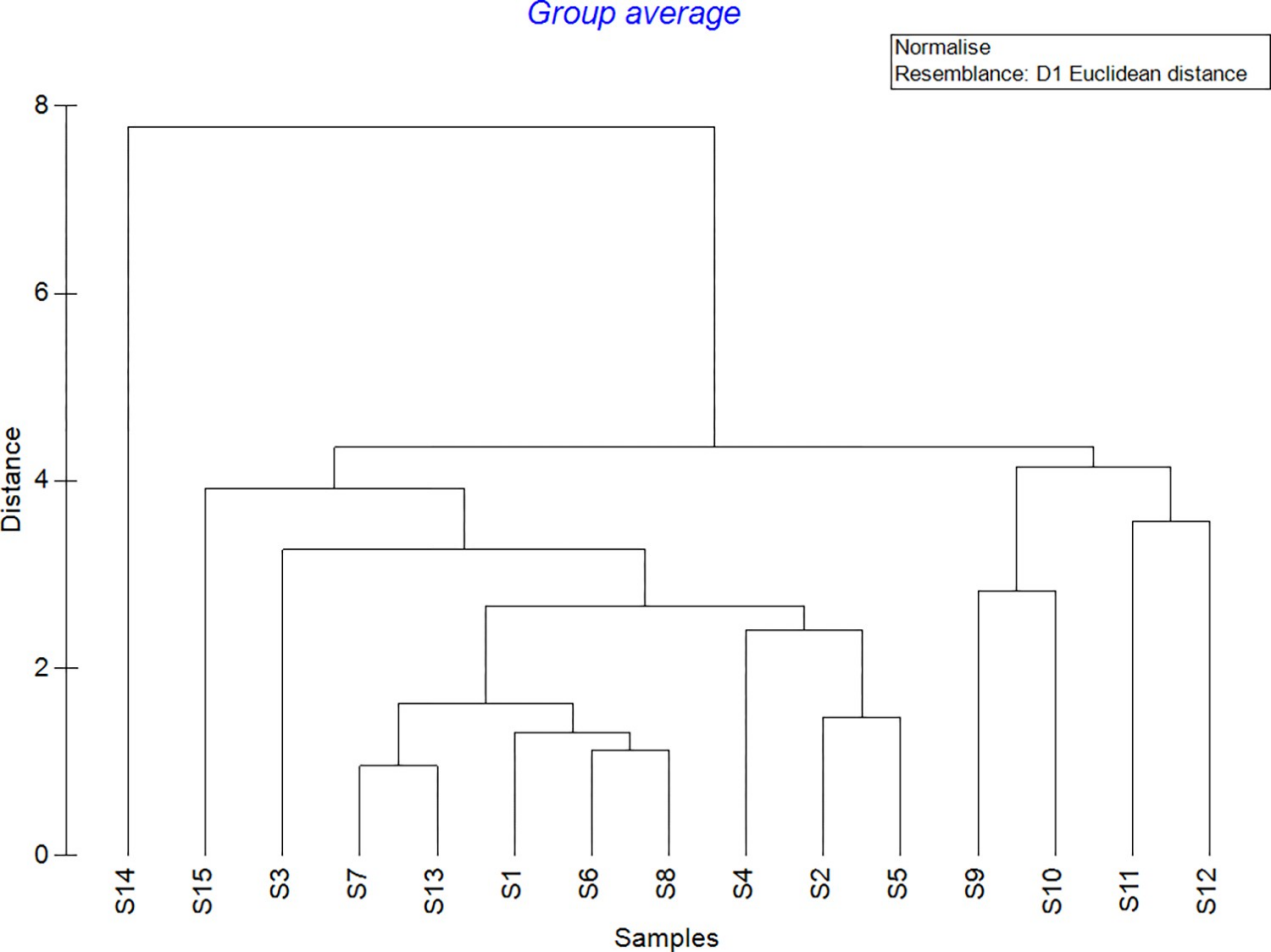
CLUSTER analysis showed the similarity of environmental parameters between stations.

During the current investigation, a total of four *in situ* physico-chemical water parameters were collected, as well as sediment for granulometry analysis (Table 1). The similarity of environmental factors across stations was shown by CLUSTER analysis using Euclidean distance (Fig 4). The stations were grouped into four groups when the similarity distance was set at 4. Station S9 and S10 were in one group, S11 and S12 were in another, S14 was in a separate group, and the rest of the stations were in a single group. The temperatures in S9 (25.17±0.26°C) and S10 (25.33±0.52°C) were slightly higher than in other stations, while the granulometry analysis showed S9 and S10 had coarser sediment environments. S9 was dominated by coarse sand (500μm-1.0mm) at 47.08% while S10 comprised mostly medium sand (250μm-500μm) at 37.25%. On the other hand, S11 and S12 were grouped together due to their extremely low salinity (S11: 16.55±0.45ppt; S12: 16.89±0.08ppt). In addition, S14 was in an independent group because of its lowest pH (6.37±0.10), highest DO (5.93±0.05mg/L) and highest silt percentage (<63μm) at 27.79% among other stations. Results of Pearson correlation showed that nematode density was positively correlated with medium sand (r = 0.593, p = 0.02).

### Discussion

Numerous studies have established correlations between environmental variables and the formation of nematode communities [43–46]. According to the majority of research, sediment grain size is the fundamental factor influencing the abundance and variety of marine nematodes [1,47,48]. The current investigation established a positive correlation between nematode density and medium sand. S9 and S10 were classified separately in CLUSTER analysis due to their coarser sediment conditions, with S9 being predominantly coarse sand and S10 being predominantly medium sand with the highest nematode density. The finer the grain size, the greater the abundance of marine nematodes, and vice versa, since the finer grain size gives a greater total surface area for organic matter adsorption [49] and so supports higher nematode community abundances [45,50].

Although S10 had the highest nematode density, S2 in the WCJBRCA had the highest number of species, species richness, and species diversity. The Shannon-Wiener diversity index (H’), which is extensively employed to quantify benthic community diversity, may also indicate sediment condition [51]. Indexes of species diversity with a value less than 2.0 indicate low species diversity and poor sediment condition, whereas those with a value greater than 3.0 indicate the reverse [52]. In general, stations located northeast of the commercial fishing harbour (S14) have a more than 2.0 species diversity value, whereas those located southeast of the commercial fishing harbour have a value less than 2.0. On the other hand, greater nematode species diversity may suggest a diversity of food supplies, whilst lower indices may indicate a scarcity or lack of food sources diversity [45,53].

S15 and S13 had the lowest sediment condition (diversity index < 2). According to residents, S13 was once a port and is now occupied with a few residential units. As a result, local inputs such as sewage or waste disposal were anticipated. Domestic sewage may contribute to localised eutrophication [54], altering the composition of nematode assemblages by organic matter enrichment and being related with pollution [55], resulting in a lack of nematode species diversity. According to Urban-Malinga [50], species diversity often decreases with pollution, while Sahraean [56] showed decreased nematode diversity and abundance in sewage-impacted areas of the city or residential complex. On the other hand, the low diversity index in S15 could be a result of the rainy weather, along with high wave conditions during sampling. Currents caused by the wind can suspend nematodes from sediment into the water column until they reach still water [57]. Additionally, Venekey [58] indicate that meiofauna may burrow down to ten centimetres into the sediment to avoid being suspended in the water column. Moreover, vertical movement of nematodes to deeper strata is conceivable in well-aerated habitats with sediment sizes greater than 125 m (S15 were primarily composed of very coarse sand, > 1 mm) and to mitigate the possibility of erosion caused by high flow velocity [59]. However, upward migration of some species may occur as a result of resource allocation and quality [60], possibly contribute to the result of certain species domination in S15.

Additionally, S10 and S14 exhibit poor sediment condition as measured by nematode diversity values, but S9, which is located between S10 and S14, exhibits moderate sediment condition. It is believed that discharge originates at S14 and collects at S10 as a result of the breakwater (Fig 1). The breakwater may have a substantial effect on the area’s currents and wave energy [61], perhaps depositing anthropogenic contaminants in S10 and explaining the moderate sediment condition in S11. Petroleum, sewage, and biomass combustion are only a few of the significant toxins found in fishing harbours that have an effect on the nematode community structure [62,63]. Additionally, elevated PAH levels in sediments suggest a high organic matter content [64], which influences the composition of marine nematodes [53,56,65].

Maturity Index was initially proposed for the study of terrestrial and freshwater habitats, and was then extended to marine and brackish ecosystems [41,66], yet it was being used conservatively [67]. Bongers [42] distinguished colonizers or c-p 1 which are more tolerant to environmental variations while presisters or c-p 5 which are more sensitive, thus, increase in number of persisters leads to an increase in MI value. Despites that it could be a good descriptor of stress as it is influenced lesser by natural variables (e.g. sediment granulometry) than other available indices such as H’ and J, there were cases with unsatisfactory results [13,68,69]. In current study, MI values showed significant contrasting results with H’ especially in S15 which was dominant by *Eurystomina* sp. 2 (c-p 4). Semprucci *et al*. [70] reported only a slight influence of granulometry on MI and c-p, thus, giving more support to the hypothesis of higher anthropogenic activity in the area. Furthermore, persisters nematodes in high abundance may either indicates the prolonged poor conditions, leading to nematode assemblage which is well adapted to the unfavourable conditions [71,72], or its ability to profit from the organisms killed by the extreme environmental conditions [13]. Therefore, this study further supports the application of H’ particularly in marine environment and H’ has been selected for the EcoQ class definition was selected for the EcoQ class definition due to its wide application [67].

In general, the MDS results indicated a low degree of similarity across the marine nematode community studied in this study. Coral reefs dominated the first category, which included S1 and S2 in the WCJBRA. Previous research has shown that the coral reef area contains a greater number of marine nematodes, either in terms of species or densities [73], in comparison to areas surrounding those subjected to anthropogenic activities [33,34], such as S14. *Spilophorella* sp., *Viscosia* sp. and *Meyersia* sp. were the top three nematode genera discovered in S1. Marine nematode, *Spilophorella* sp., was one of the dominant species observed in the study site of Semprucci [15] which has high H’ value (3.5–4.5), indicating good sediment quality. Although S1 have fairly high species diversity index, yet the index value was slightly below 3.0 which indicated the sediment quality still under a satisfactory level due to slight disturbed by the ecotourism activities in the WCJRCA. Several studies had noted the impact of tourism and diving activities on coral ecosystem [74–78]. Inexperienced divers with low buoyancy control skill have higher physical contact rate with coral reefs compare to experienced divers causing coral breakage [79]. This scenario not only will reduce the coral biomass but also cause the injured coral to suffer from slower growth rates and become more susceptible to disease and predation [80], altering the reefs state into a less structural complex state as well as impacting the benthic assemblage [81,82], thus, the lower species diversity index in S1.

In general, for most current sites, the community structure of marine nematodes was considerably different from S12, S13 and S15. The three stations were positioned most far from WCJRCA, indicating possible substantial anthropogenic disorders in household, port operations and exposure to the hydrodynamic processes. Previous studies have shown that several species of free-living marine nematodes may be used to identify pollutant levels and habitat or sediment quality [15,23,83], different environmental impacts [28,84] as well as depth of sediment collected [45]. Contaminated site evaluation normally incorporates water and sediment assessments to evaluate the overall level of contamination, but often does not indicate the toxicity of contaminants to biota [34,85]. The study by Fischer [86] shows that species of nematode showed ability and adaptive mechanisms to handle shifting environments to reduce their detrimental impacts. Some nematodes (for example, *Enoplus brevis* and *Enoplus communis*) have shown that their unique abilities to contains pollutants like Cd, Cu, Pb and Zn in their biotope [21].

The top three nematode abundances in S12 were *Daptonema* sp., *Pomponema* sp. and *Cyatholaimus* sp., while in S13 were *Chromadorita* sp., and *Innocuonema* sp. In previous studies, *Daptonema* sp., *Sabatieria* sp., *Terschellingia* sp., *Theristus* sp., and *Paradontophora* sp., were reported to be able to indicate pollution and environmental disturbances such as heavy metals [11,22,47,63], while *Microlaimus* sp. are known to inhabit anoxic environments in the study of Nanajkar [87]. *Daptonema* sp. was also proven by Mahmoudi [63] that this species showed a positive relationship with the concentration of diesel while this species was categorized as ‘opportunistic’ nematode species and elaborate their position in marine environmental biomonitoring. Thus, the presence of *Daptonema* sp. not only suggests potential low-level diesel contamination in that area but also shows that there are minimal levels of metal pollution [88]. Nematode species, *Innocuonema* sp., were found dominant in S13 and are known for their ability to tolerate different levels and types of disturbances in the study carried out by Semprucci [89]. Although the current study collected limited information on water quality parameters, previous studies had well documented the use of marine nematodes as bioindicators that could potentially be used as guidelines to estimate the potential impact of their presence in the study area.

Both *Terschellingia* sp. and *Pomponema* sp. were recorded as dominant species from S14. The former species was reported by Sahraeian [90] at study site with high polychlorinated biphenyls (PCBs) levels, a result of zinc production smelting and electrical power generation [91]. *Terschellingia* sp. also has a positive correlation with heavy metals such as Cadmium, Colbat, Chromium, Copper, Iron, Manganese, Nickel, Vadium, and Aluminium [92]. In addition, it has been linked to organic enrichment and as an indicator of poor ecological status due to its tolerance to pollution [13,93]. The latter species was documented in a previous study by Mahmoudi [63] as *Pomponema* sp. was recorded to be significantly affected by diesel contamination but not eliminated, thus, it is considered as the diesel-sensitive nematode. On the other hand, S15 was dominated by *Eurystomina* sp. with a minority of *Axonolaimus* sp. Previously, the presence of *Axonolaimus* sp. may potentially indicate the occurrence of anthropogenic disturbance in S15 as discussed by previous research [11,63,88,94] it is commonly found in areas with metal pollution and osmoregulatory stress on account of salinity values beyond the optimum range in that area.

Furthermore, different types of organic matter allow different nematode genera and species to coexist with distinct functional feeding groups (FFG) [39,40,45]. In general, epigrowth feeder (2A) dominated FFG in our investigation, implying that benthic microalgae were the most important food source coincided with the study by Leduc and Probert in 2011. On the other hand, S15 was discovered to be dominated by predators/omnivores (2B), which were made up of *Eurystomina* sp. 2. The presence of huge volumes of specific food kinds or nutrients, such as domestic discharge from neighbouring families, restaurants, or fishing vessels that potentially accumulate in the area that favours that specific group, could suggest the dominance of a given FFG [43,47].

The population structure of free-living marine nematodes in the WCJBRA and its surrounding areas was identified in this study. *Daptonema* sp., *Innocuonema* sp., *Axonolaimus* sp., and *Pomponema* sp. were discovered as nematode genera that could be used as bioindicators in the area, however more research is needed to undertake on water and sediment analyses. All the study stations were divided into three groups based on Shannon-Weiner diversity index, nematode species composition, and FFG distribution. The first group (good condition) included S1 to S8 and S11; the second group (moderate condition) included S9, S10, and S14; and the third group (poor condition) included S12, S13, and S15.

## Conclusion

The present study only showed a positive correlation between nematode density and medium sand. This indicated the presence of confounding factors in characterising the nematode community in WCJBRA and its adjacent area. Findings on the species diversity index, together with the presence of potential bioindicator species, indicate that some stations are probably being affected by anthropogenic activities regardless of the level of disturbances. Certain genera such as *Spilophorella* sp. in the stations of GC in the present study have the potential to be introduced as indicators for a healthy environment. The presence of *Daptonema* sp., *Innocuonema* sp., *Axonolaimus* sp., and *Pomponema* sp., in S12, S13, and S15 indicated potential metal, diesel and organic contaminations corresponded to the anthropogenic activities in the area (domestic waste, diesel, antifouling coating and etc). The high abundance of *Eurystomina* sp. in S15 may be a potential bioindicator and symbolises the presence of a particular contaminant which was not measured in this study. Although data on contaminants are lacking, this research enables there to be a preliminary evaluation of the current ecological conditions in WCJBRA. It may also provide a baseline for the future monitoring in WCJBRA and for assessing long-term changes therein. Therefore, further studies on the detailed water quality profiling and nematode-pollution relationship are suggested, particularly on autecology as per se for future application in environment monitoring.
